# Optimal Oral Iron Therapy for Iron Deficiency Anemia Among US Veterans

**DOI:** 10.1001/jamanetworkopen.2024.14305

**Published:** 2024-05-31

**Authors:** Nilang Patel, Scott G. Silvey, Pradeep Arora, George M. Feldman

**Affiliations:** 1 Department of Medicine, Virginia Commonwealth University, Richmond; 2 Division of Nephrology, Central Virginia VA Healthcare System, Richmond; 3 Department of Biostatistics Virginia Commonwealth University, Richmond; 4 Department of Medicine, University of Buffalo, Buffalo, New York; 5 Division of Nephrology, VA Western New York Healthcare System, Buffalo

## Abstract

**Importance:**

Optimal oral iron supplementation strategy is unclear in patients with iron deficiency anemia (IDA) who have either normal kidney function (NKF) or chronic kidney disease (CKD).

**Objective:**

To investigate the association of different oral iron supplementation strategies with the change in hemoglobin and iron indices among patients with IDA with either NKF or CKD.

**Design, Setting, and Participants:**

This retrospective cohort study was conducted between 2009 and 2019 at nationwide Veterans Health Administration facilities. Eligible participants included veterans with IDA (defined as hemoglobin <12 g/dL and either iron saturation <20% or ferritin <50 ng/mL) who received their first outpatient prescription of oral iron. Patients were further divided into those with NKF (estimated glomerular filtration rate >60 mL/min/1.73 m^2^) and CKD (estimated glomerular filtration rate ≥15 mL/min/1.73 m^2^ and <60 mL/min/1.73 m^2^). Data analysis was conducted from February to October 2023.

**Exposures:**

Patients were classified into 3 groups based on their oral iron dosing schedule: daily (once a day), multiple doses per day (MDD; ≥2 times per day), or alternate-day dose (ADD).

**Main Outcomes and Measures:**

The primary outcomes were change of hemoglobin, ferritin, total iron binding capacity (TIBC), and iron saturation (ISAT), which were calculated with linear mixed-effects models.

**Results:**

A total of 71 677 veterans with IDA (63 202 male [88.2%] and 8475 female [11.8%]; mean [SD] age, 68.47 [13.09] years), including 47 201 with NKF and 24 476 with CKD, were identifed. In patients with NKF in the daily group, hemoglobin increased from baseline (estimated per-30-day difference [SE], 0.27 [0.00] g/dL; *P* < .001). In comparison with the daily group, hemoglobin increased more in the MDD group (estimated per-30-day difference [SE], 0.08 [0.03] g/dL; *P* < .001), but no difference was noted in the ADD group (estimated per-30-day difference [SE], −0.01 [0.01] g/dL; *P* = .38). Ferritin, ISAT, and TIBC results were similar, except TIBC showed less change in the ADD group compared with the daily group. Patients with CKD showed similar trends but smaller magnitudes in changes. Among patients with NKF, the adjusted mean increase in hemoglobin was 1.03 g/dL (95% CI, 1.01-1.06 g/dL) for those in the daily group, 1.38 g/dL (95% CI, 1.36-1.40 g/dL) for those in the MDD group, and 0.93 g/dL (95% CI, 0.84-1.02 g/dL) for those in the ADD group at 90 days. Among patients with CKD, the adjusted mean increase in hemoglobin was 0.71 g/dL (95% CI, 0.68-0.73 g/dL) for those in the daily group, 0.99 g/dL (95% CI, 0.97-1.01 g/dL) for those in the MDD group, and 0.62 g/dL (95% CI, 0.52-0.73 g/dL) for those in the ADD group at 90 days.

**Conclusions and Relevance:**

In this retrospective cohort study of veterans with IDA, there was no significant difference in the improvement of hemoglobin and iron indices between daily and ADD groups, but quickest improvement was observed in the MDD group. These findings suggest that the choice of oral iron therapy should depend on the rapidity of response desired and patient preference due to adverse effects.

## Key Points

**Question:**

What is the preferred oral iron supplementation strategy in patients with iron deficiency anemia (IDA)?

**Findings:**

In this cohort study of 71 677 patients with IDA, improvement of hemoglobin and other iron indices were not different between patients who were prescribed daily or alternate day oral iron supplementation, although improvement happened at a slower pace than those who were prescribed multiple doses per day (≥2 times per day). Patients with chronic kidney disease showed similar trends but smaller magnitudes in changes compared with patients with normal kidney function.

**Meaning:**

In this cohort study, all different oral iron strategies improved hemoglobin and iron indices but at slower paces in alternate day dosing; these findings suggest that oral iron regimens should be based on patient preference and desired rapidity of response.

## Teaser

This cohort study analyzes the optimal oral iron dosing regimen for patients with iron deficiency anemia who were treated in Veterans Health Administration facilities and who had either normal kidney function or chronic kidney disease.

## Introduction

Oral iron supplementation is a preferred approach to treat iron deficiency anemia (IDA). However, iron absorption from supplements in iron-depleted patients is low (2%-13% with food and 5%-28% without food).^1^ To compensate for this low absorption and to increase bioavailability, large iron doses are often administered in divided doses, typically 2 to 3 times per day.^2,3,4^

Hepcidin is the central regulatory molecule in iron metabolism.^5^ Oral iron supplements acutely increase serum hepcidin level, and a higher level of hepcidin inhibits further absorption of iron from the gut.^6^ Hence, acute hepcidin rise triggered by a morning iron dose might lead to poor absorption of an afternoon or evening dose of oral iron.^6^ Furthermore, hepcidin levels are elevated in patients with chronic kidney disease (CKD) and may impair the absorption of oral iron and reduce the efficacy of oral iron supplementation in patients with IDA and CKD.^7,8^ However, the Kidney Disease Improving Global Outcomes guideline recommends trials of oral iron 2 to 3 times per day in patients with IDA.^9^

A recent study^10^ showed no difference in fractional oral iron absorption between single daily doses vs divided daily doses in iron deficient patients without anemia. Furthermore, compared with daily iron dosing, alternate-day iron dosing showed 34% higher rate of iron absorption. However, the association of this less frequent iron supplementation with hemoglobin and iron indices has not been properly investigated among patients with IDA who have either normal kidney function (NKF) or CKD. We investigated the association of 2 to 3 times a day oral iron administration (typical clinical practice) vs once daily oral iron administration vs alternate day oral iron administration with the change of hemoglobin and iron indices in patients with NKF and CKD.

## Methods

This cohort study was approved by the institutional review board of the Central Virginia US Department of Veterans Affairs Healthcare system. A waiver of informed consent was granted because the study was a retrospective analysis. The study followed the Strengthening the Reporting of Observational Studies in Epidemiology (STROBE) reporting guideline.

### Data Collection

Using the Veterans Health Administration Corporate Data Warehouse, we identified patients with IDA who received their first outpatient oral iron prescription (324 mg or 325mg of ferrous sulfate, ferrous gluconate, or ferrous fumarate) for 90 days, with at least 1 refill within 120 days of the first index prescription date between 2009 and 2019. IDA was defined as hemoglobin less than 12 g/dL (to convert to grams per liter, multiply by 10) and either (1) iron saturation (ISAT) less than 20% or (2) ferritin less than 50 ng/mL (to convert to micrograms per liter, multiply by 1). There was no official clinical practice guideline by the Veterans Health Administration on management of IDA, and oral iron was prescribed as per treating physician clinical discretion. Patients were classified into 3 dose groups: daily (once a day), multiple doses per day (MDD; ≥2 times per day), and alternate day dose (ADD) based on their oral iron dosing schedule.

Baseline laboratory data consisted of data collected closest to the index prescription date, but not older than 180 days. Follow-up laboratory data were collected from 30 to 180 days following the first oral iron prescription date (index prescription date). We have only included patients who had at least 1 follow-up hemoglobin measurement during this time. Ferritin, total iron binding capacity (TIBC; calculated in micrograms per deciliter [to convert to micromoles per liter, multiply by 0.179]), and ISAT were collected in those who met the aforementioned inclusion criteria. Therefore, every patient in the final cohort had continuous hemoglobin data, and within these patients, a subset had follow-up data on the other 3 iron indices.

Baseline estimated glomerular filtration rates (eGFR)^11^ were collected. Patients with an eGFR less than 15 mL/min/1.73 m^2^, patients receiving dialysis, and patients with kidney transplants were excluded. Patients were further classified based on baseline eGFR into the cohort with NKF (eGFR ≥ 60 mL/min/1.73 m^2^) and the cohort with CKD (eGFR ≥ 15 and <60 mL/min/1.73 m^2^).

Baseline age, sex, and body mass index (BMI; calculated as weight in kilograms divided by height in meters squared) and smoking status were collected. Age and BMI were converted into binary variables, with the cutoff points being 60 years and a BMI of 25.

We collected data on baseline comorbidities, Charlson Comorbidity Index (CCI), CKD staging in those with eGFR less than 60 mL/min/1.73 m^2^, diabetes, heart failure, sleep apnea, and chronic obstructive pulmonary disease (COPD). CCI was categorized as either mild (≤3) or moderate to severe (>3).

Outpatient prescriptions were also examined for the use of angiotensin converting enzyme (ACE) inhibitors, angiotensin receptor blockers (ARB), antiplatelet drugs, proton-pump inhibitors (PPI), and anticoagulants. Additionally, we examined proportions of patients receiving ferrous sulfate vs other types of oral iron (ferrous gluconate or fumarate). Patients who received intravenous iron, erythropoietin stimulating agents (ESA; eg, epoetin alfa or darbepoetin), blood transfusion, or had severe anemia with baseline hemoglobin less than 7 g/dL were excluded from the final analysis.

### Statistical Analysis

#### Cohort Characteristics

Baseline characteristics were described for each iron dose group, and within each CKD stratum. Categorical variables were presented as raw counts and percentages of the total. We used Pearson χ^2^ tests to compare characteristics between groups.

#### Laboratory Outcomes

We used linear mixed-effects models to assess the association of iron dose group (daily use was considered the reference group) with the change in hemoglobin, TIBC, ferritin, and ISAT during the follow-up period. To account for within-patient variation, a random patient-specific intercept was included. Time in days was modeled as a continuous variable, and we incorporated an autoregressive correlation structure to model the within-patient correlations between measurements at different days. Our main outcome of interest was the interaction between dose group and time. Specifically, we aimed to make inferences about the outcome of MDD and ADD iron regimens as compared with those receiving daily oral iron. We performed this mixed-model analysis on both the NKF stratum and the CKD stratum separately. Additionally, we used the output from the fitted models to calculate estimated 90-day and 180-day mean changes in each iron dose group.

We compared results from unadjusted models and models controlling for all covariates that were significantly different between the iron dose groups. As a sensitivity analysis, we added baseline hemoglobin to the mixed-effects models in order to examine any difference in results that may have arisen from baseline severity of anemia between the groups.

RStudio version 4.1.2. (Posit) was used for all statistical analyses. All hypothesis tests conducted were 2-sided, with statistical significance defined as *P* < .05. Data analysis occurred from February to October 2023.

## Results

**Figure 1.**
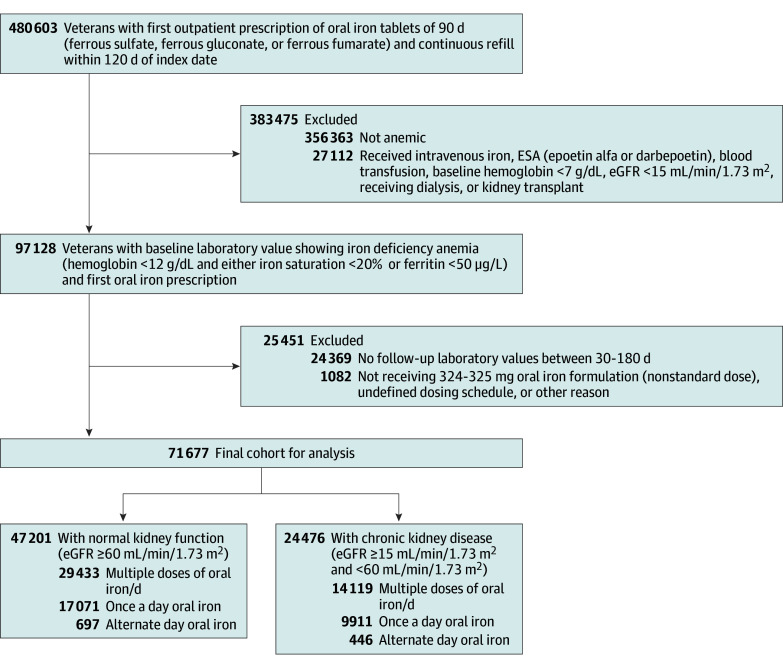
Flowchart of Study Participants To convert ferritin to micrograms per liter, multiply by 1; hemoglobin to grams per liter, multiply by 10. eGFR indicates estimated glomerular filtration rate; ESA, erythropoietin stimulating agents.

We identified 71 677 veterans (mean [SD] age, 68.47 [13.09] years; 63 202 male [88.2%] and 8475 female [11.8%]) who had IDA with first outpatient oral iron prescription with follow-up hemoglobin values between 30 and 180 days after the oral iron prescription date, including 47 201 patients with NKF and 24 476 patients with CKD (Table 1). A total of 59 983 participants (83.7%) were prescribed ferrous sulfate while 11 694 participants (16.3%) were prescribed ferrous glucoate or fumarate. Within the entire cohort, 33 764 patients (47.1%) had follow-up ferritin data, 24 018 (33.5%) had ISAT data, and 28 233 (39.4%) had TIBC data available. Figure 1 shows a detailed summary of the cohort build up.

**Table 1.** Baseline Characteristics

Characteristics	Participants by iron dosage, No (%) (N = 71 677)			*P* value
	Daily (n = 26 982)	Multiple doses per day (n = 43 552)	Alternate day dose (n = 1143)	
Cohort with normal kidney function (N = 47 201)^a^				
Participants	17 071 (63.3)	29 433 (67.6)	697 (61.0)	NA
Sex				
Male	14 322 (83.9)	24 935 (84.7)	601 (86.2)	.03
Female	2749 (16.1)	4498 (15.3)	96 (13.8)	
Age >60 y	12 511 (73.3)	20 678 (70.3)	548 (78.6)	<.001
Body mass index >25^b^	12 437 (72.9)	22 240 (75.6)	514 (73.7)	<.001
CCI score >3	4846 (28.4)	7433 (25.3)	260 (37.3)	<.001
ACE-I or ARB	7467 (43.7)	13 027 (44.3)	296 (42.5)	.39
PPI	5432 (31.8)	9851 (33.5)	222 (31.9)	.001
Anticoagulant	4017 (23.5)	5754 (19.5)	182 (26.1)	<.001
Antiplatelet	4092 (24.0)	6265 (21.3)	184 (26.4)	<.001
Iron formulation: ferrous sulfate	14 156 (82.9)	24 888 (84.6)	605 (86.8)	<.001
Diabetes	7996 (46.8)	13 244 (45.0)	334 (47.9)	<.001
COPD	5209 (30.5)	8219 (27.9)	218 (31.3)	<.001
Heart failure	4692 (27.5)	8005 (27.2)	160 (23.0)	.03
Sleep apnea	3277 (19.2)	4521 (15.4)	211 (30.3)	<.001
Current or former smoker	8902 (52.1)	15 101 (51.3)	396 (56.8)	.005
Cohort with CKD (N = 24 476)^a^				
Participants	9911 (36.7)	14 119 (32.4)	446 (39.0)	NA
Sex				
Male	9445 (95.3)	13 473 (95.4)	426 (95.5)	.89
Female	466 (4.7)	646 (4.6)	20 (4.5)	
Age >60 y	9234 (93.1)	12 887 (91.3)	417 (93.5)	<.001
Body mass index >25^b^	7305 (73.8)	10 810 (76.6)	331 (74.2)	<.001
CCI score >3	5996 (60.5)	8107 (57.4)	296 (66.4)	<.001
CKD Stage				
3A and 3B	8433 (85.1)	12 060 (85.4)	361 (80.9)	.03
4	1478 (14.9)	2059 (14.6)	85 (19.1)	
ACE-I or ARB	5171 (52.2)	7689 (54.5)	227 (50.9)	.001
PPI	2871 (29.0)	4166 (29.5)	127 (28.5)	.62
Anticoagulant	3219 (32.5)	4202 (29.8)	149 (33.4)	<.001
Antiplatelet	3116 (31.4)	3937 (27.9)	152 (34.1)	<.001
Iron formulation: ferrous sulfate	8189 (82.6)	11 747 (83.2)	398 (89.2)	.001
Diabetes	5968 (60.2)	8553 (60.6)	273 (61.2)	.81
COPD	3443 (34.7)	4681 (33.2)	138 (30.9)	.02
Heart failure	4665 (47.1)	6759 (47.9)	198 (44.4)	.20
Sleep apnea	2002 (20.2)	2532 (17.9)	131 (29.4)	<.001
Current or former smoker	4824 (48.7)	7010 (49.6)	253 (56.7)	.002

Abbreviations: ACE-I, angiotensin converting enzyme inhibitors; ARB, angiotensin receptor blockers; CCI, Charlson Comorbidity Index; CKD, chronic kidney disease; COPD, chronic obstructive pulmonary disease; NA, not applicable; PPI, proton-pump inhibitors.

^a^ Percentages in the participants rows are based on totals in each iron dosage group. All other percentages are based on the totals in the participants rows for the 2 cohorts.

^b^ Body mass index was calculated as weight in kilograms divided by height in meters squared and contained missing values (21 for the cohort with normal kidney function and 11 for the cohort with CKD). Only complete cases were considered.

### Cohort Characteristics

#### Cohort With NKF 

In the cohort of 47 201 patients with NKF (mean [SD] age, 65.31 [13.22] years; 39 858 male [84.4%] and 7343 female [15.6%]), most patients were prescribed an MDD regimen (29 433 participants [62.3%]), followed by a daily (once a day) regimen (17 071 participants [36.2%]), and an ADD regimen (697 participants [1.5%]). All characteristics except rates of ACE inhibitor and ARB medication were significantly different between the dose groups (Table 1). In general, patients prescribed MDD tended to be younger, have fewer comorbidities, have a higher BMI, were more likely to be receiving PPI, and were less likely to be receiving anticoagulants or antiplatelets compared with other dose groups. Those in the ADD group had a higher level of comorbidities except for heart failure, were older, were more likely to be male and taking ferrous sulfate, were current or former smokers, and were more likely to be receiving anticoagulants and antiplatelets compared with other groups.

#### Cohort With CKD 

In the cohort of 24 476 patients with CKD (mean [SD] age, 74.57 [10.42] years; 23 344 male [95.4%] and 1132 female [4.6%]), most patients had CKD stage 3 (20 854 patients [85.2%]). As seen in the cohort with NKF, most patients were prescribed an MDD regimen (14 119 patients [57.7%]), followed by a daily (once a day) regimen (9911 patients [40.5%]), and an ADD regimen (446 patients [1.8%]). Characteristics were similar to patients with NKF and are shown in Table 1.

### Slope of Hemoglobin and Iron Indices

#### Cohort With NKF

In patients with NKF, a daily regimen was associated with significant increase of hemoglobin over time compared with baseline (estimated per-30-day difference [SE], 0.27 [0.00] g/dL; *P* < .001). In comparison with a daily regimen, an MDD regimen was associated with an increasing slope of hemoglobin change (estimated per-30-day difference [SE], 0.08 [0.03] g/dL; *P* < .001), while the ADD regimen showed no change in trajectory when compared with the daily regimen (estimated per-30-day difference [SE], −0.01 [0.01] g/dL; *P* = .38) (Table 2).

**Table 2.** Slopes of Change in Hemoglobin and Iron Indices

Model	Slope estimate (SE)^a^	*P* value
Cohort with normal kidney function^b^		
Hemoglobin, unadjusted g/dL		
Daily	0.27 (0.00)	<.001
MDD vs daily	0.08 (0.03)	<.001
ADD vs daily	−0.01 (0.01)	.38
Hemoglobin, adjusted g/dL		
Daily	0.27 (0.00)	<.001
MDD vs daily	0.08 (0.00)	<.001
ADD vs daily	−0.01 (0.01)	.37
Ferritin, unadjusted ng/mL		
Daily	3.07 (0.30)	<.001
MDD vs daily	3.04 (0.37)	<.001
ADD vs daily	−2.70 (1.43)	.06
Ferritin, adjusted ng/mL		
Daily	3.07 (0.30)	<.001
MDD vs daily	3.03 (0.37)	<.001
ADD vs daily	−2.70 (1.43)	.06
TIBC, unadjusted µg/dL		
Daily	−6.77 (0.24)	<.001
MDD vs daily	−5.09 (0.31)	<.001
ADD vs daily	3.69 (1.19)	.002
TIBC, adjusted µg/dL		
Daily	−6.79 (0.24)	<.001
MDD vs daily	−5.06 (0.31)	<.001
ADD vs daily	3.68 (1.19)	.002
ISAT, unadjusted %		
Daily	1.97 (0.05)	<.001
MDD vs daily	0.52 (0.06)	<.001
ADD vs daily	−0.27 (0.22)	.21
ISAT, adjusted %		
Daily	1.97 (0.05)	<.001
MDD vs daily	0.52 (0.06)	<.001
ADD vs daily	−0.26 (0.22)	.24
Cohort with chronic kidney disease^c^		
Hemoglobin, unadjusted g/dL		
Daily	0.19 (0.00)	<.001
MDD vs daily	0.06 (0.00)	<.001
ADD vs daily	−0.03 (0.01)	.02
Hemoglobin, adjusted g/dL		
Daily	0.19 (0.00)	<.001
MDD vs daily	0.06 (0.00)	<.001
ADD vs daily	−0.03(0.14)	.02
Ferritin, unadjusted ng/mL		
Daily	3.11 (0.47)	**<**.001
MDD vs daily	3.36 (0.61)	<.001
ADD vs daily	−1.41 (2.13)	.51
Ferritin, adjusted ng/mL		
Daily	3.11 (0.47)	<.001
MDD vs daily	3.36 (0.61)	<.001
ADD vs daily	−1.37 (2.13)	.52
TIBC, unadjusted µg/dL		
Daily	−5.48 (0.28)	<.001
MDD vs daily	−3.85 (0.37)	<.001
ADD vs daily	2.23 (1.16)	.06
TIBC, adjusted µg/dL		
Daily	−5.50 (0.28)	<.001
MDD vs daily	−3.86 (0.37)	<.001
ADD vs daily	2.17 (1.16)	.06
ISAT, unadjusted %		
Daily	1.72 (0.05)	<.001
MDD vs daily	0.43 (0.07)	<.001
ADD vs daily	0.02 (0.21)	.91
ISAT, adjusted %		
Daily	1.73 (0.05)	<.001
MDD vs daily	0.43 (0.07)	<.001
ADD vs daily	0.04 (0.21)	.83

Abbreviations: ADD, alternate day dose; ISAT, iron saturation; MDD, multiple doses per day; TIBC, total iron binding capacity.

Conversion factors: To convert ferritin to micrograms per liter, multiply by 1; hemoglobin to grams per liter, multiply by 10; TIBC to micromoles per liter, multiply by 0.179.

^a^ Slopes represent the 30-day change for each laboratory value.

^b^ Cohort with normal kidney function was adjusted for Charslon Comorbidity Index, body mass index, age, sex, diabetes, chronic obstructive pulmonary disease, anticoagulants, antiplatelets, proton-pump inhibitors, type of oral iron, sleep apnea, and smoking status.

^c^ Cohort with chronic kidney disease was adjusted for Charlson Comorbidity Index, body mass index, age, chronic obstructive pulmonary disease, anticoagulants, antiplatelets, type of oral iron, angiotensin converting enzymes and angiotensin receptor blockers, current or former smoking, chronic kidney disease staging, and sleep apnea.

Ferritin and ISAT results were similar. Specifically, a daily regimen was associated with significant increases over time compared with baseline ferritin (estimated per-30-day difference [SE], 3.07 [0.30] ng/mL; *P* < .001) and ISAT (estimated per-30-day difference [SE], 1.97% [0.05%]; *P* < .001). In comparison with a daily regimen, an MDD regimen was associated with a significantly steeper increase in ferritin (estimated per-30-day difference [SE], 3.04 [0.37] ng/mL; *P* < .001) and ISAT (estimated per-30-day difference [SE], 0.52% [0.06%]; *P* < .001), while an ADD regimen did not show differences in slopes in ferritin (estimated per-30-day difference [SE], −2.70 [1.43] ng/mL; *P* = .06) and ISAT (estimated per-30-day difference [SE], −0.27% [0.22%]; *P* = .21).

For TIBC, a daily regimen was associated with a decrease over time (estimated per-30-day difference [SE], −6.77 [0.24] μg/dL; *P* < .001). In comparison with a daily regimen, an MDD regimen was associated with a further decline in TIBC slope (estimated per-30-day difference [SE], −5.09 [0.31] μg/dL; *P* < .001), while an ADD regimen showed an increase in slope (estimated per-30-day difference [SE], 3.69 [1.19] μg/dL; *P* = .002). After adjusting for CCI, BMI, age, sex, diabetes, COPD, anticoagulants, antiplatelets, PPI, type of oral iron, sleep apnea, and smoking status, the statistical significance of these results did not change (Table 2).

#### Cohort With CKD

Among patients with CKD, a daily regimen was associated with a significant increase in hemoglobin over time compared with baseline (estimated per-30-day difference [SE], 0.19 [0.00] g/dL; *P* < .001). Compared with a daily regimen, hemoglobin increased more in with an MDD regimen (estimated per-30-day difference [SE], 0.06 [0.00] g/dL; *P* < .001) and increased less with an ADD regimen (estimated per-30-day difference [SE], −0.03 [0.01] g/dL; *P* = .02).

Ferritin increased over time in the daily group (estimated per-30-day difference [SE], 3.11 [0.47] ng/mL; *P* < .001). Compared with a daily regimen, ferritin increased more with an MDD regimen (estimated per-30-day difference [SE], 3.36 [0.61] ng/mL; *P* < .001) but did not differ with an ADD regimen (estimated per-30-day difference [SE], −1.41 [2.13] ng/mL; *P* = .51). TIBC and ISAT results were similar and are shown in Table 2. After adjusting for CCI, BMI, age, COPD, anticoagulants, antiplatelets, type of oral iron, ACE and ARB, current or former smoking, CKD staging, and sleep apnea, the statistical significance of these results did not change (Table 2).

### Sensitivity Analysis

Mean (SD) hemoglobin at baseline was significantly different between the 3 dose groups in both the NKF cohort (daily, 10.61 [1.05] g/dL; MDD, 10.36 [1.14] g/dL; ADD, 10.44 [1.10] g/dL; *P* < .001) and CKD cohort (daily, 10.43 [1.10] g/dL; MDD, 10.22 [1.11] g/dL; ADD, 10.35 [1.10] g/dL; *P* < .001), although the absolute differences were small (eTable 1 in Supplement 1). To control for the potential confounding effect of lower baseline hemoglobin on the improvement of hemoglobin, we added baseline hemoglobin as an additional covariate in these models. Results did not change (eTable 2 and eTable 3 in Supplement 1).

### Estimates of Change in Hemoglobin and Iron Indices

#### Cohort With NKF

**Figure 2.**
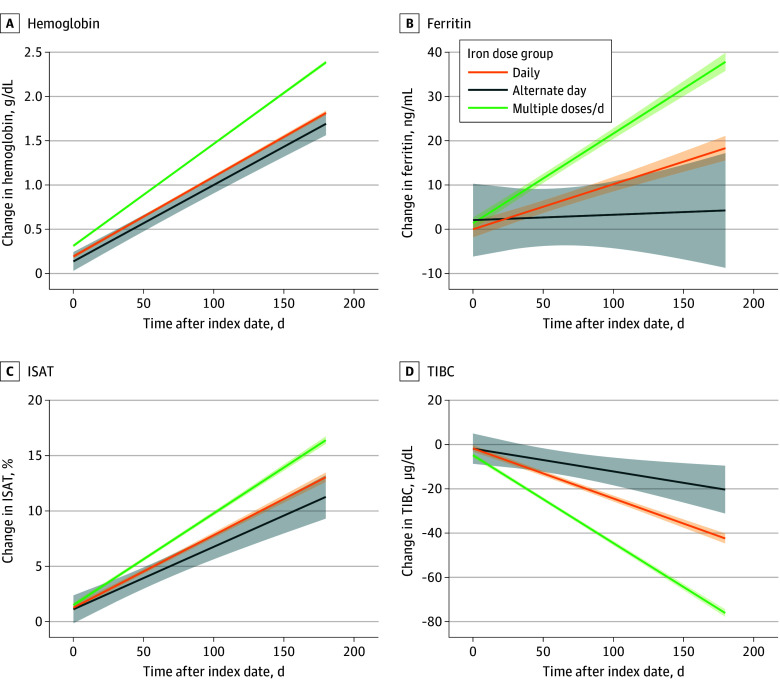
Estimated Change in Hemoglobin and Iron Indices in Cohort With Normal Kidney Function Figure shows the mean rates of change in hemoglobin (A), ferritin (B), ISAT (C), and TIBC (D) among patients with iron deficiency anemia and normal kidney function in each dose group up to 180 days postindex date. Lines represent estimates from linear mixed-effects models, and the shaded regions represent 95% CIs. Plots correspond to models without added covariates for adjustment because these did not affect estimates in a meaningful manner. To convert ferritin to micrograms per liter, multiply by 1; hemoglobin to grams per liter, multiply by 10; TIBC to micromoles per liter, multiply by 0.179. ISAT indicates iron saturation; TIBC, total iron binding capacity.

Figure 2 shows the mean rates of change in hemoglobin and iron indices in the NKF cohort for each dose group as obtained from the linear mixed-effects models. As observed previously, an MDD regimen was associated with larger changes in the slope of hemoglobin and iron indices as compared with a daily regimen. An ADD regimen was not associated with differences in rates of change of hemoglobin, ferritin, and ISAT for those in the daily group. The trajectory of TIBC for the ADD group was less steep than the daily group. Adjustment of covariates did not change estimates in a meaningful way.

Table 3 shows the mean estimated changes and 95% CIs for hemoglobin and iron indices of each dose group at 90 and 180 days (adjusted model). At 90 days, the associated mean increase in hemoglobin was 1.38 g/dL (95% CI, 1.36-1.40 g/dL) in the MDD group, 1.03 g/dL (95% CI, 1.01-1.06) in the daily group, and 0.93 g/dL (95% CI, 0.84-1.02) in the ADD group. At 180 days, the mean increase in hemoglobin was 2.42 g/dL (95% CI, 2.39-2.44 g/dL) in the MDD group, 1.85 g/dL (95% CI, 1.82-1.88 g/dL) in the daily group, and 1.71 g/dL (95% CI, 1.58-1.84) in the ADD group. Similar trends were seen in ferritin, TIBC, and ISAT at 90 days and 180 days (Table 3).

**Table 3.** Estimates of Change in Hemoglobin and Iron Indices at 90 and 180 Days (Adjusted Model)

Time point	Dosage group, mean change (95% CI)		
	Daily	Multiple doses per day	Alternate day dose
Cohort with normal kidney function^a^			
90 d			
Hemoglobin, g/dL	1.03 (1.01 to 1.06)	1.38 (1.36 to 1.40)	0.93 (0.84 to 1.02)
Ferritin, ng/mL	9.86 (7.89 to 11.82)	20.39 (18.74 to 22.03)	3.49 (−3.77 to 10.75)
TIBC, µg/dL	−24.07 (−25.65 to −22.50)	−42.05 (−43.39 to −40.71)	−12.72 (−18.64 to −6.80)
ISAT, %	7.64 (7.34 to 7.93)	9.43 (9.18 to 9.69)	6.55 (5.48 to 7.62)
180 d			
Hemoglobin, g/dL	1.85 (1.82 to 1.88)	2.42 (2.39 to 2.44)	1.71 (1.58 to 1.84)
Ferritin, ng/mL	19.07 (16.03 to 22.10)	38.69 (36.33 to 41.05)	4.59 (−8.49 to 17.67)
TIBC, µg/dL	−44.45 (−46.91 to −42.00)	−77.62 (−79.58 to −75.67)	−22.05 (−32.86 to −11.24)
ISAT, %	13.55 (13.08 to 14.02)	16.89 (16.51 to 17.27)	11.68 (9.71 to 13.65)
Cohort with chronic kidney disease^b^			
90 d			
Hemoglobin, g/dL	0.71 (0.68 to 0.73)	0.99 (0.97 to 1.01)	0.62 (0.52 to 0.73)
Ferritin, ng/mL	10.01 (7.05 to 12.96)	21.21 (18.73 to 23.68)	7.82 (−3.54 to 19.17)
TIBC, µg/dL	−17.21 (−18.80 to −15.62)	−31.32 (−32.67 to −29.97)	−11.64 (−17.46 to −5.81)
ISAT, %	6.35 (6.09 to 6.62)	7.76 (7.53 to 8.00)	6.86 (5.91 to 7.81)
180 d			
Hemoglobin, g/dL	1.27 (1.24 to 1.31)	1.75 (1.72 to 1.77)	1.09 (0.95 to 1.23)
Ferritin, ng/mL	19.34 (14.68 to 23.99)	40.63 (36.78 to 44.47)	13.05 (−6.21 to 32.30)
TIBC, µg/dL	−33.71 (−36.39 to −31.04)	−59.40 (−61.63 to −57.17)	−21.64 (−31.84 to −11.44)
ISAT, %	11.54 (11.07 to 12.01)	14.24 (13.83 to 14.65)	12.17 (10.43 to 13.91)

Abbreviations: ISAT, iron saturation; TIBC, total iron binding capacity.

Conversion factors: To convert ferritin to micrograms per liter, multiply by 1; hemoglobin to grams per liter, multiply by 10; TIBC to micromoles per liter, multiply by 0.179.

^a^ Cohort with normal kidney function was adjusted for Charslon Comorbidity Index, body mass index, age, sex, diabetes, chronic obstructive pulmonary disease, anticoagulants, antiplatelets, proton-pump inhibitors, type of oral iron, sleep apnea, and smoking status.

^b^ Cohort with chronic kidney disease was adjusted for Charlson Comorbidity Index, body mass index, age, chronic obstructive pulmonary disease, anticoagulants, antiplatelets, type of oral iron, angiotensin converting enzymes and angiotensin receptor blockers, current or former smoking, chronic kidney disease staging, and sleep apnea.

#### Cohort With CKD

Like Figure 2, the eFigure in Supplement 1 shows mean rates of change in hemoglobin and iron indices in patients with CKD. At 90 days, the associated mean increase in hemoglobin was 0.99 g/dL (95% CI, 0.97-1.01 g/dL) in the MDD group, 0.71 g/dL (95% CI, 0.68-0.73 g/dL) in the daily group, and 0.62 g/dL (95% CI, 0.52-0.73 g/dL) in the ADD group. Similar trends were observed in the change of hemoglobin and other iron indices at 180 days. (Table 3).

## Discussion

In this large, retrospective, observational cohort study of veterans with IDA, we found that an MDD regimen of oral iron supplementation was associated with significant improvement in hemoglobin compared with a daily or ADD oral iron supplementation regimen. The difference in the improvement of hemoglobin between the daily and the ADD oral iron supplementation strategies was not significant for patients with NKF, but there was a slight difference for patients with CKD. However, this difference had little clinical significance for the improvement of hemoglobin at 90 and 180 days. Patients with CKD had lower improvement in hemoglobin and iron indices compared with patients with NKF across the different dose groups.

Earlier reports^12,13^ suggested that intermittent oral iron supplementation may be equally effective as daily oral iron in pregnant and young menstruating women. A recent study^10^ using radioactively labeled oral iron showed that alternate-day oral iron supplementation resulted in better iron absorption and a lower rise in serum hepcidin compared with patients who received divided doses of oral iron per day. Although increased oral iron absorption theoretically should lead to higher hemoglobin levels, this study^10^ did not focus on hemoglobin change. Study participants received oral iron doses after an overnight fast and fasted for 3 hours after the dose. They had a total of 60 healthy, White, young, iron deficient women without anemia, obesity, and comorbidities.^10^ Because of its small sample size, short duration (approximately 28 days), and without a meaningful outcome like change in hemoglobin, it is difficult to extrapolate findings^10^ to clinical settings.

Our study results differ substantially from what was published by others.^6,10^ We had a large sample size and studied changes in hemoglobin up to 6 months. Although we do not have a clear explanation for our findings, hepcidin dynamics with oral iron have previously been studied mainly with healthy young females. Our patients, on the other hand, were older males with IDA and multiple comorbidities. It is possible that our patients might have had slightly higher baseline serum hepcidin levels and may not have experienced similar hepcidin dynamics with additional oral iron dosing as others have shown, and therefore responded better to frequent dosing.

Our study results are in line with other small studies. A randomized clinical trial^14^ of 62 young patients with IDA showed that more patients achieved 2 g/dL rise in hemoglobin in a twice a day, 60 mg, elemental oral iron regimen compared with an ADD, 120 mg, elemental oral iron regimen at 3 weeks and 6 weeks. Similarly, in another small study of 200 patients,^15^ equivalent dose of oral iron supplementation (120 mg alternate day vs 60 mg daily) did not show any difference in mean change in hemoglobin or serum ferritin at 8 weeks between ADD vs daily regimen. In our study, there was no evidence of a difference in improvement of hemoglobin between daily vs ADD oral iron strategy at 90 and 180 days.

Our study has several strengths. We have a large sample size, a longer duration of follow-up, and have included patients with IDA who were prescribed commonly used oral iron prescription for 90 days with confirmed refill between 90 and120 days, suggesting ongoing compliance with oral iron tablets. We have studied clinically meaningful outcomes like change in hemoglobin with different dosing strategies. We have done sensitivity analysis to show that baseline hemoglobin level does not affect the result. Although, iron absorption from the different ferrous formulation was about the same and mainly affected by dietary habits, to account for any variation in absorption, we have adjusted our model with different iron formulations and PPI.^1,16,17^ Because decreased kidney function is known to affect oral iron absorption, we have studied patients with CKD as a separate subcohort.

### Limitations

Our study has several limitations. First, because of the retrospective observational nature of the study, there may be a possibility of unadjusted confounding variables affecting the results. Second, we do not have patient-level malabsorption, gastrointestinal adverse effects, and tolerability data. Third, our study population is primarily older male veterans with a higher disease burden, so the result cannot be generalized to pregnant or younger patients with IDA. Fourth, we have assumed that patients were compliant and were taking what was prescribed, however, some noncompliance is possible. If so, this should have affected the MDD regimen the most, although results of the study speak otherwise and give more validity to our results.

## Conclusions

This cohort study signifies the importance of following the standard clinical practice (2-3 times a day oral iron supplementation) in IDA when a rapid rise of hemoglobin is desired. There was no evidence of a difference in improvement of hemoglobin and other iron indices between the daily and ADD oral iron strategy, and improvement happened at a slower pace than the MDD oral iron strategy. Patients with CKD showed similar responses with different oral iron dosing strategies; however, the magnitude of the changes was smaller compared with those with NKF. The choice of oral iron therapy should depend on the rapidity of response desired and patient preference due to adverse effects.

## Notes

Supplement 1. **eTable 1.** Baseline Hemoglobin **eTable 2.** Slopes of Change in Hemoglobin After Adjusting Model With Baseline Hgb **eTable 3.** Estimates of Change in Hemoglobin at 90 and 180 Days (Adjusted Model) **eFigure.** Estimate Change in Hemoglobin and Iron Indices in Cohort With Chronic Kidney Disease (CKD)

Supplement 2.
Data Sharing Statement
