# Supplementary material for: Optimal Oral Iron Therapy for Iron Deficiency Anemia Among US Veterans
Source: JAMA Netw Open. 2024 May 31;7(5):e2414305. doi: 10.1001/jamanetworkopen.2024.14305 (PMC11143460; doi:10.1001/jamanetworkopen.2024.14305)
Supplement: Supplement 1. — eTable 1. Baseline Hemoglobin eTable 2. Slopes of Change in Hemoglobin After Adjusting Model With Baseline Hgb eTable 3. Estimates of Change in Hemoglobin at 90 and 180 Days (Adjusted Model) eFigure. Estimate Change in Hemoglobin and Iron Indices in Cohort With Chronic Kidney Disease (CKD) [file jamanetwopen-e2414305-s001.pdf]

Supplemental Online Content

Patel N, Silvey SG, Arora P, Feldman GM. Optimal oral iron therapy in iron deficiency anemia among US veterans. *JAMA Netw Open.* 2024;7(5):e2414305. doi:10.1001/jamanetworkopen.2024.14305

eTable 1. Baseline Hemoglobin

eTable 2. Slopes of Change in Hemoglobin After Adjusting Model With Baseline Hgb

eTable 3. Estimates of Change in Hemoglobin at 90 and 180 Days (Adjusted Model)

eFigure. Estimate Change in Hemoglobin and Iron Indices in Cohort With Chronic Kidney Disease (CKD)

This supplemental material has been provided by the authors to give readers additional information about their work.

© 2024 Patel N et al. *JAMA Network Open.*

eTable 1: Baseline Hemoglobin

Mean (SD) Baseline Hemoglobin (g/dl)*				
	**Daily**	**MDD**	**ADD**	***p*-values**
**NKF**	10.61 (1.05)	10.36 (1.14)	10.44 (1.10)	< 0.001
**CKD**	10.43 (1.10)	10.22 (1.11)	10.35 (1.10)	< 0.001

***Although there is significant different between the three dose groups in both cohorts, the absolute differences were small.*

eTable 2: Slopes of change in Hemoglobin after adjusting model with baseline Hgb

Per-30-day slopes **(**Adjusted models w/all covariates + baseline hemoglobin)

**Cohort with NKF**		
**Model**	**Slope Estimate (s.e.)**	***p*-values**
HGB, adj.	Daily vs. baseline 0.268 (0.00257)	**<0.001**
	MDD vs. Daily : 0.0751 (0.00323)	**<0.001**
	ADD vs. Daily : -0.0123 (0.0128)	0.34
**Cohort with CKD**		
**Model**	**Slope Estimate (s.e.)**	***p*-values**
HGB, adj.	Daily vs. baseline 0.187 (0.00295)	**<0.001**
	MDD vs. Daily : 0.0615 (0.00385)	**<0.001**
	ADD vs. Daily : -0.0318 (0.0140)	**0.02**

© 2024 Patel N et al. *JAMA Network Open.*

eTable 3: Estimates of change in Hemoglobin at 90 and 180 days (adjusted model)

(Adjusted models w/all covariates + baseline hemoglobin)

	**Cohort with NKF**		
**Timepoint**	**Daily Mean (95% CI)**	**Multi-Dose per Day (MDD) Mean (95% CI)**	**Alternate Day Dose (ADD) Mean (95% CI)**
**90 Days**			
Hgb	1.12 [1.10-1.15]	1.36 [1.34-1.38]	0.95 [0.86-1.04]
**180 Days**			
Hgb	1.93 [1.90-1.96]	2.39 [2.37-2.42]	1.72 [1.59-1.84]
	**Cohort with CKD**		
**Timepoint**	**Daily Mean (95% CI)**	**Multi-Dose per Day (MDD) Mean (95% CI)**	**Alternate Day Dose (ADD) Mean (95% CI)**
**90 Days**			
Hgb	0.80 [0.77-0.82]	0.99 [0.97-1.01]	0.69 [0.59-0.79]
**180 Days**			
Hgb	1.36 [1.33-1.39]	1.74 [1.71-1.77]	1.15 [1.01-1.29]

© 2024 Patel N et al. *JAMA Network Open.*

eFigure. Estimate Change in Hemoglobin and Iron Indices in Cohort With Chronic Kidney Disease (CKD)

eFigure 1: Estimate change in Hemoglobin and Iron Indices in Cohort with Chronic Kidney Disease (CKD); Figure shows change in a). Hemoglobin b). Ferritin c). ISAT d). TIBC

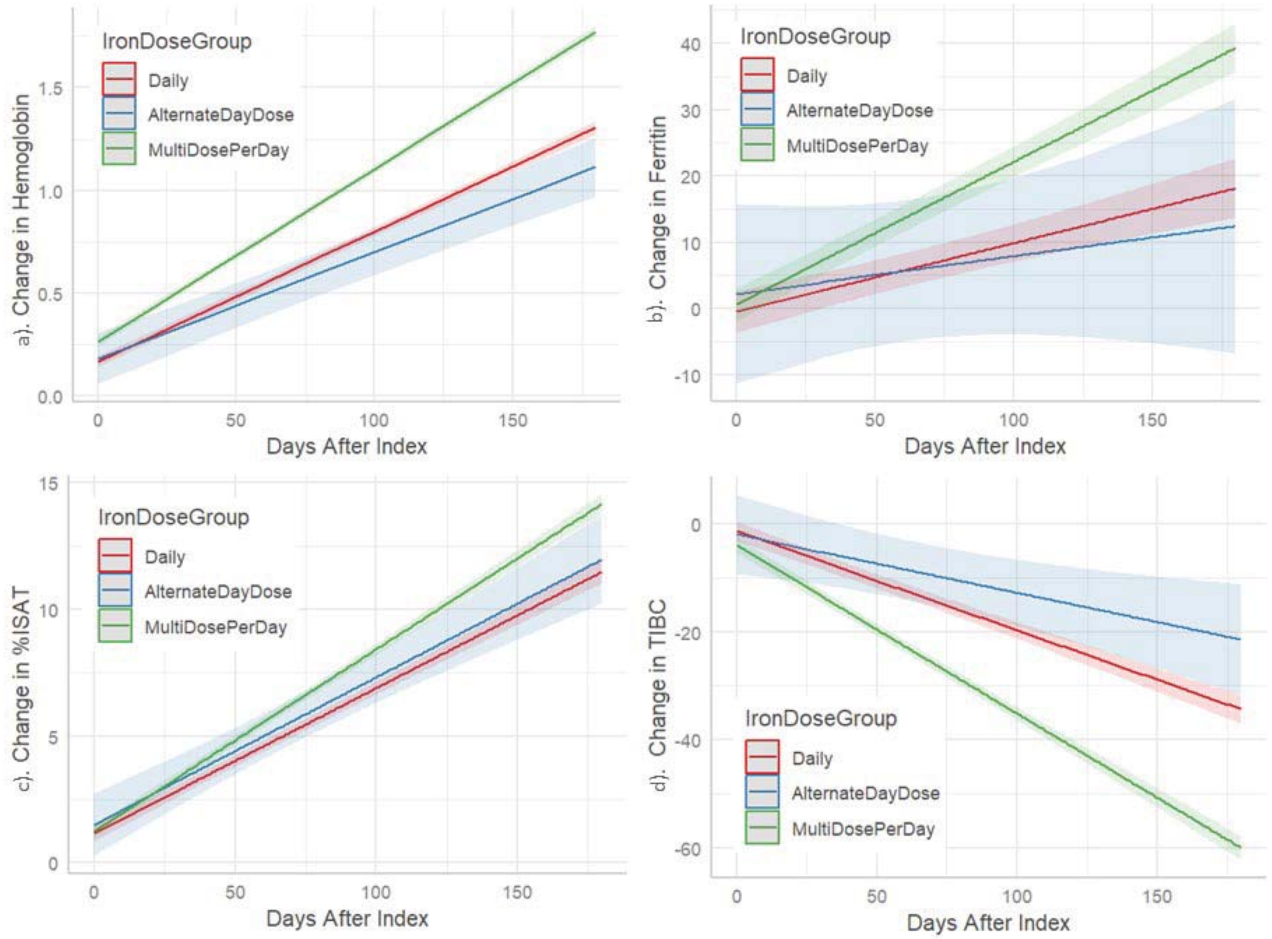

Mean rates of change in hemoglobin and iron indices among patients with IDA with CKD, in each dose group up to 180 days post-index. Lines represent estimates from linear mixed-effects models, and the shaded regions represent 95% confidence intervals. Plots correspond to models without added covariates for adjustment, as these did not affect estimates in a meaningful manner.

© 2024 Patel N et al. *JAMA Network Open.*
