# Supplementary material for: Optimal Oral Iron Therapy for Iron Deficiency Anemia Among US Veterans
Source: JAMA Netw Open. 2024 May 31;7(5):e2414305. doi: 10.1001/jamanetworkopen.2024.14305 (PMC11143460; doi:10.1001/jamanetworkopen.2024.14305)
Supplement: Supplement 2. — Data Sharing Statement [file jamanetwopen-e2414305-s002.pdf]

Data Sharing Statement

Patel. Optimal Oral Iron Therapy for Iron Deficiency Anemia Among US Veterans. *JAMA Netw Open*. Published May 31, 2024. doi:10.1001/jamanetworkopen.2024.14305

Data
Data available: No

Additional Information
Explanation for why data not available: Our study data is the property of Veterans Health Administration, US government. So, we can not share the data.
